# Gastrin‐Releasing Peptide Facilitates Cholinergically Mediated Contractions of the Mouse Gastric Fundus

**DOI:** 10.1002/prp2.70151

**Published:** 2025-07-18

**Authors:** Diego Currò, Raj Makwana, Gareth J. Sanger

**Affiliations:** ^1^ Blizard Institute Queen Mary University of London London UK; ^2^ Dipartimento di Sicurezza e Bioetica, Sezione di Farmacologia Università Cattolica del Sacro Cuore ‐ Fondazione Policlinico Universitario A. Gemelli IRCCS Roma Italia

**Keywords:** acetylcholine, bombesin, gastric fundus, gastrin‐releasing peptide, neuromedin B, stomach

## Abstract

Gastrin‐releasing peptide (GRP) and neuromedin B (NMB) are found in the stomach's myenteric plexus and muscular layers. When exogenously administered, they contract stomach muscle in several species, but their ability to modulate enteric nerve functions has rarely been studied. We investigated the effects of GRP on cholinergic‐mediated contractions of the stomach, after first characterizing the contractile effects of GRP, NMB, and bombesin (BB). Circular muscle rings, cut from the mouse gastric fundus, were studied using tissue bath techniques. Contractions evoked by electrical field stimulation (EFS; 5 Hz) were examined under non‐adrenergic, non‐nitrergic conditions. Contractions were expressed as % of the maximum contraction induced by carbachol (100 μM). GRP (0.0001–1 μM), NMB (0.0001–3 μM), and BB (0.0001–1 μM) induced concentration‐dependent tonic muscle contractions. The *p*D_2_ and *E*_*max*_ were, respectively, GRP: 7.2 ± 0.1, 54.6% ± 3.6% (*n* = 14); BB: 7.4 ± 0.2, 47.2% ± 3.9% (*n* = 9); and NMB: 6.3 ± 0.1, 38.1% ± 5.6% (*n* = 10). GRP‐ and NMB‐induced contractions were inhibited by the BB_2_ receptor antagonist BW‐10, not by the BB_1_ receptor antagonist PD168368. EFS‐evoked contractions were blocked by atropine (1 μM) or tetrodotoxin (1 μM), and concentration‐dependently potentiated by GRP (0.0001–1 μM), with *p*D_2_ and *E*_*max*_ values 7.8 ± 0.1 and 81.2% ± 14.0%, respectively (*n* = 17). This activity was inhibited by BW‐10, not PD168368. BW‐10 alone also reduced EFS‐evoked contraction amplitudes. GRP and BW‐10 did not affect contractions induced by bethanechol (1–300 μM). In conclusion, in mouse gastric fundus, GRP causes muscle contraction and also facilitates electrically evoked cholinergic activity (facilitating ACh release), both actions most likely involving BB_2_ receptor activation. Further experiments must ascertain the physiological significance of these actions, alone and together.

## Graphical abstract

Gastrin‐releasing peptide concentration‐dependently potentiates the cholinergic contractions of mouse gastric fundus circular muscle evoked by electric field stimulation.

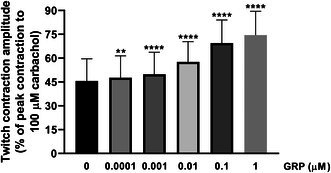

**Abbreviations**
BB: bombesin
EFS: electrical field stimulation
ENS: enteric nervous system
GI: gastrointestinal
GRP: gastrin‐releasing peptide
NMB: neuromedin B
NO: nitric oxide
TTX: tetrodotoxin

## 1 Introduction

The bombesin (BB) receptor family comprises three G_q/11_ protein‐coupled receptor subtypes (BB_1_, BB_2_, and BB_3_ receptors) and takes its name from a 14‐amino‐acid peptide isolated from the skin of *Bombina bombina* and *Bombina variegata* frogs that acts as an agonist at these receptors [1]. In mammals, the endogenous ligands of the BB_1_ and BB_2_ receptors, gastrin‐releasing peptide (GRP) and neuromedin B (NMB), were discovered a few years after BB [2, 3]. GRP and NMB are 27‐ and 10‐amino‐acid peptides, respectively. GRP can be further processed to the carboxy‐terminal decapeptide GRP_18–27_ (also known as neuromedin C [4]). At the human receptors, GRP has approximately 779 times higher affinity for BB_2_ than for BB_1_, while NMB has approximately 673 times higher affinity for BB_1_ than for BB_2_ [4]. The endogenous ligand of BB_3_ receptors is still unknown; GRP and NMB have a low affinity for human BB_3_ receptors (affinity constants > 3 μM [4]).

Within the gastrointestinal (GI) tract of various mammalian species, GRP and NMB mRNA and immunoreactivity have been found in myenteric neurons of the stomach and both myenteric and submucosal neurons of the intestine [4, 5, 6, 7]. Also within the stomach and intestine of different mammalian species, BB receptors have been identified within the circular and longitudinal muscle layers, in addition to the cell bodies and nerve fibers of the myenteric plexus, and the cell bodies of the interstitial cells of Cajal [4, 8, 9, 10, 11, 12, 13]. In terms of function, most studies with isolated GI tissues show an ability of exogenous GRP, NMB, and BB to directly cause contraction of the smooth muscle and/or facilitate ongoing spontaneous contractile activity, usually without involving neuronal activity [4, 14]. For example, in different regions of the human stomach, BB caused tonic muscle contractions that were unaffected by atropine or tetrodotoxin (TTX) and greater in amplitude than those caused by acetylcholine [15]. It has been suggested that contraction of the gastroduodenal junction explains how BB receptor agonists reduce gastric emptying of solid or liquid meals in human volunteers and rats, following systemic administration [16, 17, 18, 19, 20]. Nevertheless, a smaller number of studies have also demonstrated the ability of BB receptor agonists to stimulate enteric nerve functions. For example, in guinea pig isolated ileum, the application of BB and GRP depolarized myenteric AH/Type 2 neurons [21]. Such observations may be consistent with the expression of BB receptors within the enteric nervous system (ENS; see above), but the physiological functions of enteric nerve stimulation are unclear.

In the present study, we compared the ability of GRP to contract mouse isolated stomach muscle and also facilitate cholinergic‐mediated contractions, characterizing the BB receptors mediating each function. The aim was to clarify the actions of GRP on gastric neuromuscular functions. The purpose was to help understand the seemingly anomalous reports of an ability of subcutaneously administered BB to increase gastric emptying of a liquid meal in rats [22] and the reported mixed ability of GRP and BB to inhibit and then, after prolonged intravenous infusion, stimulate gastric emptying [23].

## 2 Methods

### 2.1 Policy

This paper complies with the Uniform Requirements for Manuscripts Submitted to Biomedical Journals of the International Committee of Medical Journals Editors (ICMJE).

### 2.2 General Methods

For this study, 35 virgin female (mean weight 26.6 ± 0.5 g) and 36 male (mean weight 30.5 ± 0.3 g) CD1 mice were used as described in detail elsewhere [24]. Briefly, mice were purchased from Charles River, UK, and housed in a room in the university animal unit with a controlled temperature (22°C ± 1°C), humidity (55% ± 10%), and 12‐h light–dark cycle. Males and females were kept in separate cages, with food (LabDiet EURodent diet 14%, International Product Supplies Ltd., London, UK) and water provided ad libitum until euthanasia by cervical dislocation followed by exsanguination to ensure death under ASPA Schedule 1.

The stomachs were removed and placed in room temperature Krebs solution of the following composition (mM): NaCl 118.3, KCl 4.7, CaCl_2_ 2.5, KH_2_PO_4_ 1.2, MgSO_4_ 1.2, NaHCO_3_ 25, and glucose 11.1 (pH 7.4). The gastric fundus was then separated from the rest of the stomach, carefully cleaned of its contents, and two circular muscle rings were prepared, mounted between platinum wire electrodes connected to STG2008 stimulators (Multi Channel Systems, Germany), and suspended in Krebs solution bubbled with a 95%/5% O_2_/CO_2_ mixture and maintained at 37°C inside 5‐ or 10‐ml tissue baths. The rings were connected to isometric force transducers (MLT201/D, ADInstruments, UK) and stretched until a tension of 9.8 milliNewtons (mN) developed. Muscle activity (mN) was recorded using the AcqKnowledge v3.8.1 data acquisition system (BIOPAC Systems, USA) on personal computers (www.dell.com/uk). EFS, consisting of rectangular and bipolar pulses of 110% supramaximal voltage and constant duration (0.5 ms), was applied through the platinum electrodes connected to STG2008 stimulators as trains of 5 Hz frequency and 10 s duration, every 1 min, to obtain regular and uniform contractions. At the beginning of the experiments, the tissues were allowed to equilibrate in Krebs solution for 60 min. The bathing Krebs solution was renewed every 15 (5‐ml baths) or 30 (10‐ml baths) min (during the equilibration period and, where applicable, between drug administration and/or EFS periods).

### 2.3 Experimental Series

In a first series of experiments, the preparations were subjected to successive cumulative incubations with increasing concentrations of GRP (0.1 nM–1 μM), NMB (0.1 nM–3 μM), or BB (0.1 nM–1 μM). Peak contractions were obtained at each concentration of these pharmacological agents. Carbachol (100 μM) was then added to the bathing solution to obtain maximal contractions. As for the cumulative concentration–response curves to GRP and NMB, one of the two rings obtained from each gastric fundus served as a time‐matched vehicle control, and the other served in parallel to test the effects of the BB_1_ receptor antagonist PD168368 (0.1 μM) [25, 26] or the BB_2_ receptor antagonist BW‐10 (1 μM; also named BW2258U89) [27] on GRP‐ and NMB‐induced contractions.

In a second series of experiments, the rings were studied under non‐adrenergic, non‐nitrergic conditions to remove any adrenergic inhibitory prejunctional modulation of acetylcholine release and the ability of nitric oxide (NO) to inhibit neuromuscular functions. In this way, we minimized potentially confounding issues caused by simultaneous activation of excitatory and inhibitory neurotransmission and optimized the ability of EFS to evoke nerve‐mediated contractions of the muscle. For this purpose, the bath medium contained the NO synthase inhibitor N^G^‐nitro‐L‐arginine methyl ester (L‐NAME; 300 μM), the selective α‐adrenergic receptor antagonist phentolamine (0.3 μM), and the selective β‐adrenergic receptor antagonist propranolol (0.1 μM). Once reproducible and stable contractions were obtained in response to repeated EFS, the effects of TTX (1 μM), atropine (1 μM), or successive cumulative incubations with increasing concentrations of GRP (0.1 nM–1 μM) were studied. In this series of experiments, the effects induced by GRP (0.1 nM–1 μM) were also investigated in separate groups of preparations pre‐treated with PD168368 (0.1 μM) or BW‐10 (1 μM). Receptor antagonists were always pre‐incubated for 15 min.

To assess whether facilitation of the EFS‐evoked contractions by GRP was caused simply by its ability to increase muscle tone, additional experiments were performed to look for changes in the response to EFS when muscle tone was increased by a non‐BB receptor agonist. For this purpose, we used successive cumulative additions of increasing concentrations of the prostanoid TP receptor agonist 9,11‐dideoxy‐9α,11α‐methanoepoxy prostaglandin F_2α_ (U46619; 10 nM–1 μM).

To verify whether the effects of GRP and BW‐10 on EFS‐induced contractions were due to pre‐ or postjunctional activities, low concentrations of GRP (10 nM) or BW‐10 (1 μM) were investigated for any ability to modulate the cumulative concentration‐response curve to bethanechol (1–300 μM).

### 2.4 Statistical Analysis

Contractions evoked by the different receptor agonists were calculated as peak amplitudes and are expressed as percentages of the maximal contractions induced by carbachol (100 μM). From these data, *p*D_2_s were determined as the cologarithms of half maximal effective concentrations (EC_50_s), and efficacies (*E*_*max*_) as the % of the maximum contraction induced by carbachol (100 μM). In the experiments with EFS, the contraction evoked by EFS just before addition of a ligand was used as the control, against which the maximum effect of that treatment was calculated as a percentage change. Results between tissues were evaluated using Student's paired or unpaired t‐test. When more than two groups were compared, analysis of variance (one‐way ANOVA) followed by Tukey's or Dunnett's test for multiple comparisons was used. All values are presented as mean ± SEM; *p* < 0.05 was considered statistically significant. The GraphPad Prism program (GraphPad Software, San Diego, CA, USA) was used to fit concentration‐response curves and calculate EC_50_s and maximum effects.

### 2.5 Drugs

The following were used: bethanechol chloride (Thermo Fisher Scientific, Waltham, MA, USA); BW‐10 (Bachem, Bubendorf, Switzerland); carbamylcholine (carbachol) chloride (Acros Organics, Thermo Fisher Scientific, Waltham, MA, USA); atropine, phentolamine hydrochloride, tetrodotoxin citrate (Sigma‐Aldrich, St Louis, MO, USA); human GRP, propranolol hydrochloride, U46619 (Tocris, Bristol, UK); NMB, bombesin, L‐NAME (Alfa Aesar, Thermo Fisher Scientific, Waltham, MA, USA); PD168368 (Cayman Chemical, Ann Arbor, MI, USA). The substances were dissolved in bi‐distilled water, except for PD168368, which was dissolved in DMSO at 10 mM, and U46619, which was delivered predissolved in methyl acetate at 10 mg ml^−1^. The latter two compounds were then diluted with bi‐distilled water. The final concentrations of DMSO and methyl acetate in the bath medium did not affect the contractions evoked by EFS or the direct smooth muscle contractions caused by activation of BB receptors.

### 2.6 Nomenclature of Targets and Ligands

Key protein targets and ligands in this article are hyperlinked to corresponding entries in http://www.guidetopharmacology.org, the common portal for data from the IUPHAR/BPS Guide to Pharmacology [28], and are permanently archived in the Concise Guide to Pharmacology 2023/24 [29].

## 3 Results

### 3.1 Ability to Cause Muscle Contraction

**FIGURE 1:**
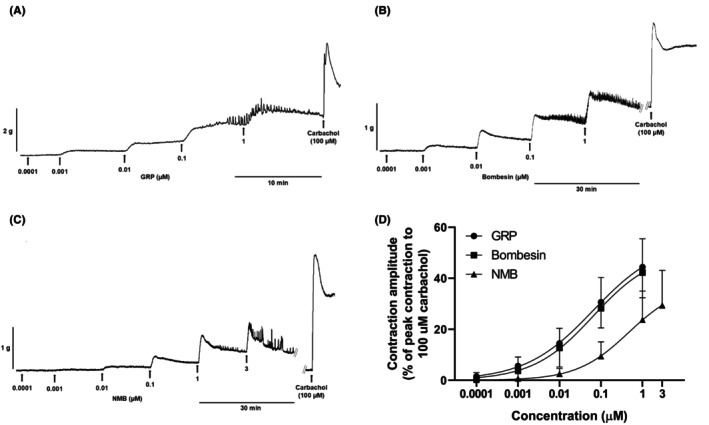
Contractions of circular muscle rings from mouse gastric fundus induced by GRP, bombesin, and NMB. A, B, C Representative traces showing the concentration‐dependent contractile effects of successive cumulative incubations with increasing concentrations of GRP (0.1 nM–1 μM) (A), bombesin (0.1 nM–1 μM) (B), and NMB (0.1 nM–3 μM) (C), respectively. The traces were obtained from different preparations. (D) Mean concentration‐response curves for contractions induced by GRP (0.1 nM–1 μM), bombesin (0.1 nM–1 μM), and NMB (0.1 nM–3 μM). Each point represents the mean ± SD of responses observed in 14, 9, and 10 rings for GRP, bombesin, and NMB, respectively. Contractile effects were calculated as peak amplitudes and are expressed as a percentage of the maximal contraction induced by carbachol (100 μM) at the end of each experiment.

GRP (0.1 nM–1 μM), NMB (0.1 nM–3 μM), and BB (0.1 nM–1 μM) induced concentration‐dependent tonic contractions of mouse gastric fundus rings (Figure 1). The contractions started with a short latency and developed in a few minutes to peak levels. These were generally well maintained at the lower concentrations, while at concentrations of 0.1–3 μM the contractions declined to lower levels (Figure 1). In addition, at the latter concentrations, irregular phasic contractions were superimposed on the tonic contractions (Figure 1). GRP and BB were approximately equipotent and similarly effective (Table 1). NMB was approximately 7.4‐fold and 10.8‐fold less potent than GRP and BB, and less effective than GRP and BB.

**TABLE 1:** *p*D_2_s and *E*_*max*_s (mean ± SEM) of concentration‐dependent contractions of the mouse gastric fundus induced by GRP, BB, and NMB.

	*p*D_2_	*E*_*max*_
GRP (*n* = 14)	7.2 ± 0.1	54.6% ± 3.6%
BB (*n* = 9)	7.4 ± 0.2	47.2% ± 3.9%
NMB (*n* = 10)	6.3 ± 0.1^a^	38.1% ± 5.6%^b^
		
GRP (*n* = 5)	7.3 ± 0.1	61.0% ± 8.2%
GRP + BW‐10 (1 μM; *n* = 5)	6.6 ± 0.1^c^	38.3% ± 6.0%^d^
		
GRP (*n* = 6)	7.2 ± 0.2	51.8% ± 4.1%
GRP + PD168368 (0.1 μM; *n* = 6)	7.5 ± 0.2	51.8% ± 5.0%
		
NMB (*n* = 5)	6.4 ± 0.05	39.3% ± 6.8%
NMB + BW‐10 (1 μM; *n* = 5)	5.7 ± 0.3^e^	11.4% ± 3.6%^f^
		
NMB (*n* = 5)	6.2 ± 0.2	36.8% ± 9.6%
NMB + PD168368 (0.1 μM; *n* = 5)	6.6 ± 0.1	30.1% ± 5.6%

*Note:* For each experiment, cumulative and increasing concentrations of GRP (0.1 nM–1 μM), NMB (0.1 nM–3 μM), or BB (0.1 nM–1 μM) were added to the bathing solution, and peak contractions were determined as a % of the contractions induced by carbachol (100 μM). To determine the effects of a receptor antagonist, one of the two rings obtained from each gastric fundus served as a time‐matched vehicle control, and the other served in parallel. Receptor antagonists were preincubated for 15 min. *p*D_2_s were determined as the cologarithms of half maximal effective concentrations (EC_50_s). Efficacies (*E*_*max*_) were determined as the % of the maximum contraction induced by carbachol (100 μM). ^a^ *p* < 0.0001 versus GRP and BB, one‐way ANOVA followed by Tukey's test for multiple comparisons; ^b^ *p* = 0.025 versus GRP, one‐way ANOVA followed by Tukey's test for multiple comparisons; ^c^ *p* = 0.008 versus GRP, Student's paired *t*‐test; ^d^ *p* = 0.016 versus GRP, Student's paired *t*‐test; ^e^ *p* = 0.04 versus NMB, Student's paired *t*‐test; and ^f^ *P* = 0.016 versus NMB, Student's paired t‐test.

**FIGURE 2:**
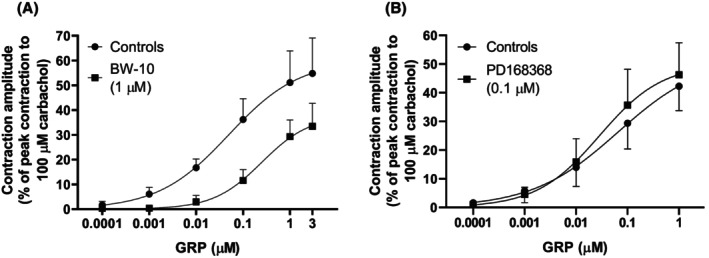
Effects of the BB_2_ receptor antagonist BW‐10 and the BB_1_ receptor antagonist PD168368 on GRP‐induced contractions of circular muscle rings from mouse proximal stomach. Mean concentration‐response curves for contractions induced by successive cumulative incubations with increasing concentrations of GRP (0.1 nM–3 μM) or (0.1 nM–1 μM), respectively, without and with BW‐10 (1 μM) (A) or PD168368 (0.1 μM) (B) in the bath medium are shown. Each point represents the mean ± SD of responses observed in five (A) or six (B) gastric fundus rings. Contractile effects were calculated as peak amplitudes and are expressed as a percentage of the maximal contraction induced by carbachol (100 μM) at the end of each experiment.

The BB_2_ receptor antagonist BW‐10 (1 μM) shifted the concentration‐response curve of GRP to the right and lowered the *E*_*max*_ (Figure 2A; Table 1). In contrast, the BB_1_ receptor antagonist PD168368 (0.1 μM) did not significantly modify either the *p*D_2_ or *E*_*max*_ of the GRP‐induced concentration‐response curve (Figure 2B; Table 1).

**FIGURE 3:**
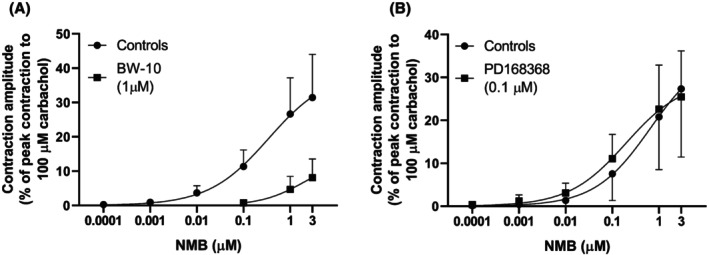
Effects of the BB_2_ receptor antagonist BW‐10 and the BB_1_ receptor antagonist PD168368 on NMB‐induced contractions of circular muscle rings from mouse proximal stomach. Mean concentration‐response curves for contractions induced by successive cumulative incubations with increasing concentrations of NMB (0.1 nM–3 μM) without and with BW‐10 (1 μM) (A) or PD168368 (0.1 μM) (B) in the bath medium are shown. Each point represents the mean ± SD of responses observed in five gastric fundus rings in both (A) and (B). Contractile effects were calculated as peak amplitudes and are expressed as a percentage of the maximal contraction induced by carbachol (100 μM) at the end of each experiment.

BW‐10 (1 μM) greatly reduced the *E*_*max*_ and decreased the *p*D_2_ of NMB (Figure 3A; Table 1). The BB_1_ receptor antagonist PD168368 (0.1 μM) did not significantly modify either the *p*D_2_ or *E*_*max*_ of the NMB‐induced concentration–response curve (Figure 3B; Table 1).

### 3.2 Effects on Neuronally‐Mediated Contractions

**FIGURE 4:**
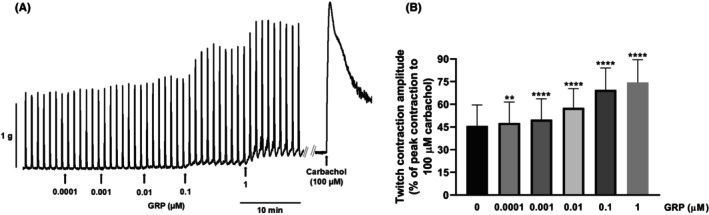
Effects of GRP on twitch contractions of circular muscle rings from mouse gastric fundus induced by EFS (0.5 ms, supramaximal voltage, 5 Hz) trains (10 s duration, delivered every 1 min) under non‐adrenergic non‐nitrergic conditions (300 μM L‐NAME plus 0.3 μM phentolamine plus 0.1 μM propranolol in the bath medium). (A) Representative trace showing the concentration‐dependent facilitatory effects of successive cumulative incubations with increasing concentrations of GRP (0.1 nM–1 μM) on EFS‐induced twitch contractions. (B) Mean EFS‐induced twitch contractions observed in the absence (0 GRP, controls) or presence of GRP (0.1 nM–1 μM). Twitch contractions were calculated as peak amplitudes and are expressed as a percentage of the maximal contraction induced by carbachol (100 μM) at the end of each experiment. The twitch contraction just before the addition of the first concentration of GRP was used as a control, and then the highest twitch contraction during the incubation of each concentration of GRP was selected for the calculations. Each column represents the mean ± SD of responses observed in 17 gastric fundus rings. Significant differences between test and control responses: **p = 0.02, ****p < 0.0001, one‐way ANOVA followed by Dunnett's test for multiple comparisons.

**FIGURE 5:**
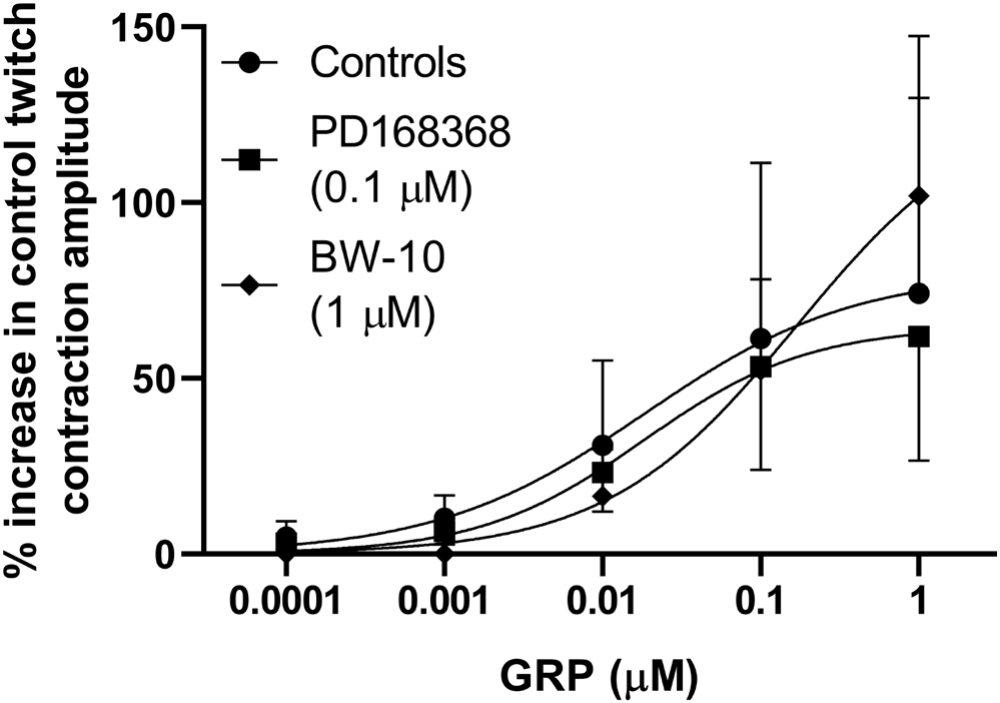
Effects of successive cumulative incubations with increasing concentrations of GRP on twitch contractions of circular muscle rings from mouse gastric fundus induced by EFS (0.5 ms, supramaximal voltage, 5 Hz) trains (10 s duration, delivered every 1 min) under non‐adrenergic non‐nitrergic conditions (300 μM L‐NAME plus 0.3 μM phentolamine plus 0.1 μM propranolol in the bath medium) in the absence or presence of the BB_1_ receptor antagonist PD168368 or the BB_2_ receptor antagonist BW‐10. Mean concentration‐response curves for the % increase in control twitch contraction amplitude induced by GRP (0.1 nM–1 μM) in the absence or presence of PD168368 (0.1 μM) or BW‐10 (1 μM) are shown. Twitch contractions were calculated as peak amplitudes. The twitch contraction just before the addition of the first concentration of GRP or GRP (10 nM) for preparations incubated with PD168368 (0.1 μM) or BW‐10 (1 μM), respectively, was used as a control, and then the highest twitch contraction during the incubation of each concentration of GRP, without or with any BB receptor antagonist in the bath, was selected for the calculations. Each point represents the mean ± SD of the responses observed in 17, 4, or 8 gastric fundus rings for GRP alone and GRP in the presence of PD168368 or BW‐10, respectively.

In non‐adrenergic non‐nitrergic conditions, EFS at 5 Hz induced reproducible contractions, whose amplitude was 51.2% ± 2.3% (*n* = 42) of the maximal contraction induced by application of carbachol (100 μM) at the end of the experiments. These contractions were rapidly abolished by TTX (1 μM; *n* = 8) and slowly reduced to 8.5% ± 2.4% of controls by atropine (1 μM, 18‐min incubation; *n* = 3). GRP (0.1 nM–1 μM) concentration‐dependently increased the EFS‐evoked contractions; the *p*D_2_ and *E*_*max*_ were, respectively, 7.8 ± 0.1 and 81.2% ± 14.0% (*n* = 17; Figures 4 and 5). Notably, the *p*D_2_ calculated from these experiments was greater than that for the concentration‐response curves for GRP‐induced muscle contractions (respectively, 7.8 ± 0.1 and 7.2 ± 0.1; *p* < 0.001).

**FIGURE 6:**
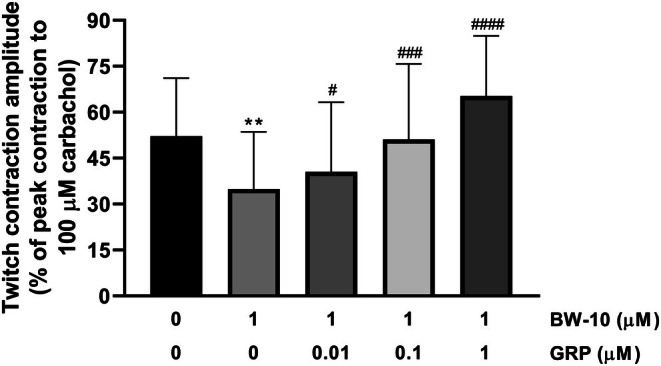
Effects of the BB_2_ receptor antagonist BW‐10 alone or successive cumulative incubations with increasing concentrations of GRP in the presence of this antagonist in the bath medium on twitch contractions of circular rings from mouse gastric fundus induced by EFS (0.5 ms, supramaximal voltage, 5 Hz) trains (10 s duration, delivered every 1 min) under non‐adrenergic non‐nitrergic conditions (300 μM L‐NAME plus 0.3 μM phentolamine plus 0.1 μM propranolol in the bath medium). Mean EFS‐induced twitch contractions were observed in the absence of any substance (0 BW‐10 and 0 GRP, controls) and in the presence of BW‐10 (1 μM) alone or with GRP (0.01–1 μM). Twitch contractions were calculated as peak amplitudes and are expressed as a percentage of the maximal contraction induced by carbachol (100 μM) at the end of each experiment. The twitch contraction just before the addition of BW‐10 was used as a control, and then the lowest twitch contraction during the incubation of BW‐10 alone and the highest twitch contraction during the incubation of each concentration of GRP with BW‐10 were selected for the calculations. Each column represents the mean ± SD of responses observed in eight gastric fundus rings. Significant differences between responses: **p = 0.003 versus controls, ^#^ *p* = 0.03, ^###^ *p* = 0.0006, ^####^ *p* < 0.0001 versus BW‐10, one‐way ANOVA followed by Dunnett's test for multiple comparisons.

**FIGURE 7:**
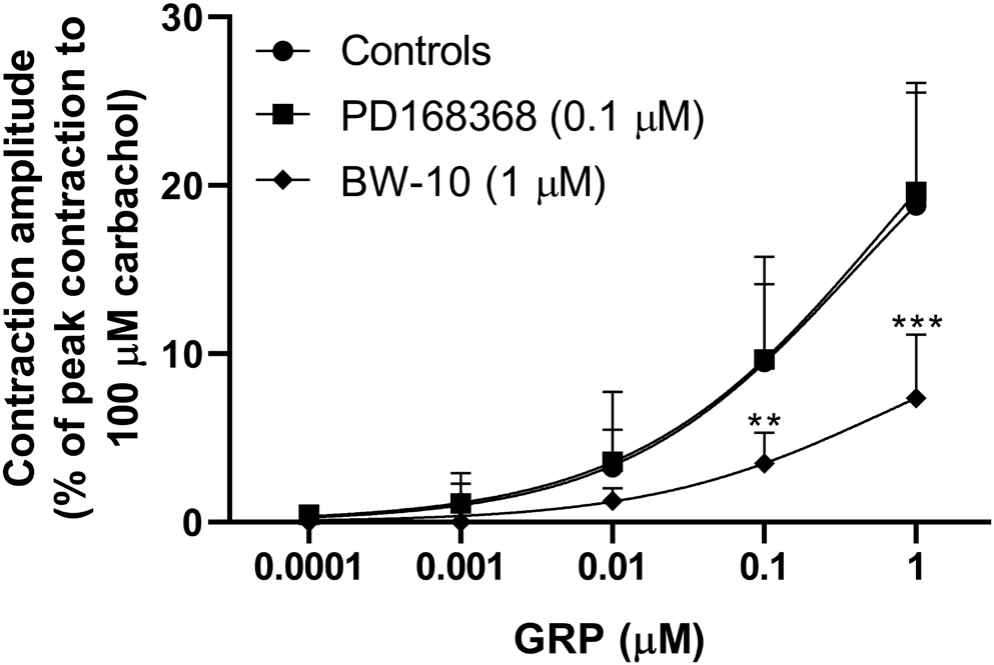
Tonic smooth muscle contractions induced by successive cumulative incubations with increasing concentrations of GRP in circular muscle rings from mouse gastric fundus subjected to EFS (0.5 ms, supramaximal voltage, 5 Hz) trains (10 s duration, delivered every 1 min) under non‐adrenergic non‐nitrergic conditions (300 μM L‐NAME plus 0.3 μM phentolamine plus 0.1 μM propranolol in the bath medium) in the absence or presence of the BB_1_ receptor antagonist PD168368 or the BB_2_ receptor antagonist BW‐10. Mean concentration‐response curves for contractions induced by GRP (0.1 nM–1 μM) in the absence or presence of PD168368 (0.1 μM) or BW‐10 (1 μM) are shown. Contractile effects were calculated as peak amplitudes and are expressed as a percentage of the maximal contraction induced by carbachol (100 μM) at the end of each experiment. Each point represents the mean ± SD of the responses observed in 17, 4, or 8 gastric fundus rings for GRP alone and GRP in the presence of PD168368 or BW‐10, respectively. Significant differences between test and control responses: **p = 0.008, ***p = 0.0003, one‐way ANOVA followed by Dunnett's test for multiple comparisons.

The BB_1_ receptor antagonist PD168368 (0.1 μM) did not significantly affect the electrically‐evoked contractions (respectively, 51.7% ± 10.1% and 52.0% ± 9.3% of the maximal contraction induced by carbachol, without and with PD168368 in the bath, *n* = 4) or the parameters of the concentration‐response curve for the facilitatory effect of GRP (in the presence of PD168368: *p*D_2_: 7.8 ± 0.06; *E*_*max*_: 64.9% ± 18.6%, *n* = 4; Figure 5). In contrast, the BB_2_ receptor antagonist BW‐10 (1 μM) reduced EFS‐evoked contractions to 64.5% ± 6.0% of controls (*n* = 8, *p* < 0.01; Figure 6). The inhibitory effect of BW‐10 developed slowly. GRP (0.1 nM–1 μM) was added when the contractions appeared stable (approximately 20 min after addition of BW‐10 in all preparations). However, during the period when the first two concentrations of GRP (0.1 and 1 nM) were added to the bathing solution, the EFS‐evoked contractions continued to decrease slightly. For this reason, the responses to 0.1 and 1 nM GRP were discounted. In the presence of BW‐10, the higher concentrations (10 nM–1 μM) of GRP increased the EFS‐evoked contractions in a concentration‐dependent manner (Figure 6), but the concentration‐response curve was now shifted to the right (Figure 5); the *p*D_2_ and *E*_*max*_ of the concentration‐response curve for GRP were 7.0 ± 0.2 (*p* < 0.001 vs. controls) and 136.8% ± 32.9% (*p* = 0.08 vs. controls). In this experimental series, GRP (0.1 nM–1 μM) also induced concentration‐dependent tonic contractions of the gastric rings that were unaffected by 0.1 μM PD168368 but significantly reduced by 1 μM BW‐10 (Figure 7).

**FIGURE 8:**
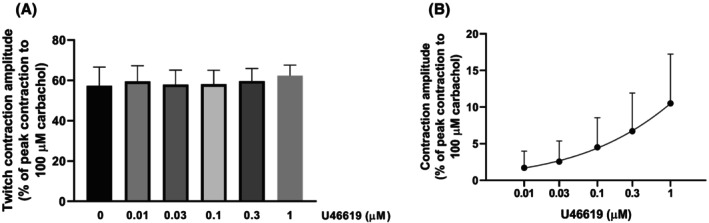
Effects of successive cumulative incubations with increasing concentrations of 9,11‐dideoxy‐9α,11α‐methanoepoxy prostaglandin F_2α_ (U46619) on circular muscle rings from mouse gastric fundus subjected to EFS (0.5 ms, supramaximal voltage, 5 Hz) trains (10 s duration, delivered every 1 min) under non‐adrenergic non‐nitrergic conditions (300 μM L‐NAME plus 0.3 μM phentolamine plus 0.1 μM propranolol in the bath medium). (A) Mean EFS‐induced twitch contractions observed in the absence (0 U46619, controls) or presence of U46619 (0.01–1 μM). Twitch contractions were calculated as peak amplitudes and are expressed as a percentage of the maximal contraction induced by carbachol (100 μM) at the end of each experiment. The twitch contraction just before the addition of the first concentration of U46619 was used as a control, and then the highest twitch contraction during the incubation of each concentration of U46619 was selected for the calculations. Each column represents the mean ± SD of responses observed in eight gastric fundus rings. (B) Mean concentration‐response curve for tonic smooth muscle contractions induced by U46619 (0.01–1 μM). Contractile effects were calculated as peak amplitudes and are expressed as a percentage of the maximal contraction induced by carbachol (100 μM) at the end of each experiment. Each point represents the mean ± SD of the responses observed in eight gastric fundus rings.

To investigate whether GRP‐induced tonic contractions of the gastric smooth muscle could be responsible for the GRP‐induced increase in the amplitude of contractions evoked by EFS, the effects of another substance that contracts the proximal gastric muscle, the TP receptor agonist U46619 [30], were investigated for any ability to increase the EFS‐evoked contractions. U46619 (10 nM–1 μM) concentration‐dependently contracted the gastric fundus rings but did not significantly affect the EFS‐evoked contractions (Figure 8).

The mean amplitudes of the control EFS‐evoked contractions in the rings exposed to GRP (0.1 nM–1 μM) alone or with PD168368 (0.1 μM), or BW‐10 (1 μM), or to U46619 (10 nM–1 μM) were not significantly different from each other (respectively, 45.6% ± 3.4% [*n* = 17], 52.0% ± 9.3% [*n* = 4], 52.2% ± 6.7% [*n* = 8], and 57.3% ± 3.3% [*n* = 8]; *p* = 0.32).

**FIGURE 9:**
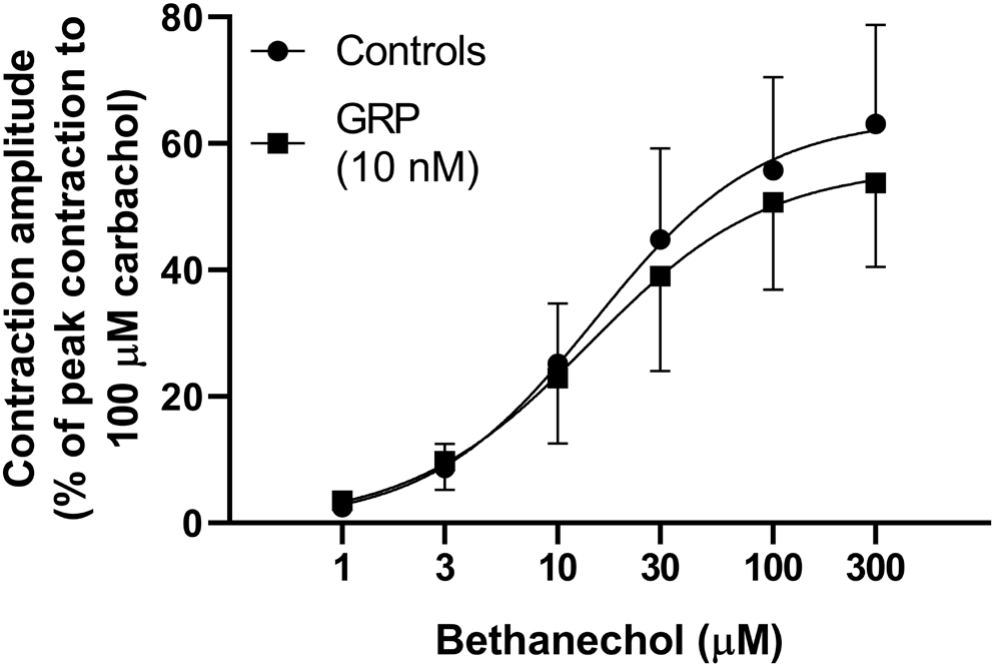
Contractions of circular muscle rings from mouse gastric fundus induced by bethanechol in the absence or presence of GRP in the bath medium. Mean concentration‐response curves for contractions induced by bethanechol (1–300 μM) without or with GRP (10 nM) in the bath medium. Each point represents the mean ± SD of responses observed in 10 rings. Contractile effects were calculated as peak amplitudes and are expressed as a percentage of the maximal contraction induced by carbachol (100 μM) at the end of each experiment.

**FIGURE 10:**
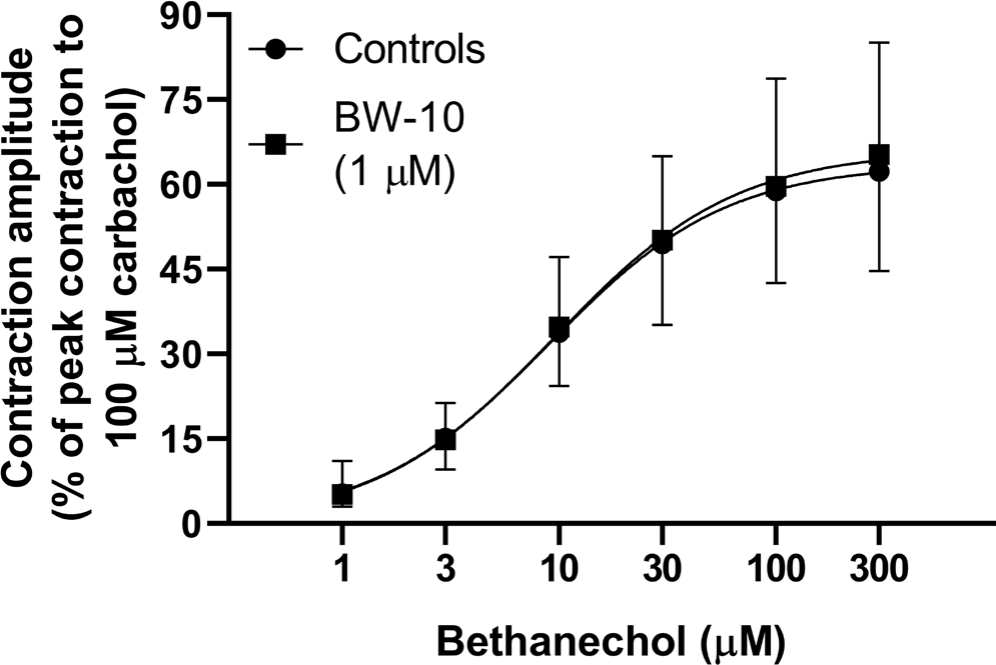
Contractions of circular muscle rings from mouse gastric fundus induced by bethanechol in the absence or presence of the BB_2_ receptor antagonist BW‐10 in the bath medium. Mean concentration‐response curves for contractions induced by bethanechol (1–300 μM) without or with BW‐10 (1 μM) in the bath medium. Each point represents the mean ± SD of responses observed in 10 rings. Contractile effects were calculated as peak amplitudes and are expressed as a percentage of the maximal contraction induced by carbachol (100 μM) at the end of each experiment.

To investigate whether the facilitatory or inhibitory effects induced respectively by GRP (0.1 nM–1 μM) and BW‐10 (1 μM) on the EFS‐evoked contractions could be due to actions on postjunctional mechanisms, the effects of GRP (10 nM; a concentration that induces a low smooth muscle contraction) and BW‐10 (1 μM) were each studied on bethanechol (1–300 μM)‐induced contractions. In these experiments, neither GRP (10 nM) nor BW‐10 (1 μM) significantly affected the concentration–response curve for bethanechol (Figures 9 and 10; Table 2).

**TABLE 2:** *p*D_2_s and *E*_*max*_s (mean ± SEM) of concentration‐dependent contractions of the mouse gastric fundus induced by bethanechol.

	*p*D_2_	*E*_*max*_
Bethanechol (*n* = 10)	4.8 ± 0.1	68.3% ± 6.1%
Bethanechol + GRP (10 nM; *n* = 10)	4.9 ± 0.1	55.9% ± 4.3%^a^
		
Bethanechol (*n* = 10)	5.1 ± 0.1	64.4% ± 6.0%
Bethanechol + BW‐10 (1 μM; *n* = 10)	5.0 ± 0.1	67.2% ± 6.1%

*Note:* For each experiment, cumulative and increasing concentrations of bethanechol (1–300 μM) were added to the bathing solution, and peak contractions were determined as a % of the contractions induced by carbachol (100 μM). To determine the effects of GRP (10 nM) or BW‐10 (1 μM), one of the two rings obtained from each gastric fundus served as a time‐matched vehicle control, and the other served in parallel. Bethanechol was added to the bathing solution of preparations incubated with GRP (10 nM) when a peak contraction to GRP was obtained. BW‐10 (1 μM) was preincubated for 15 min. *p*D_2_s were determined as the cologarithms of half maximal effective concentrations (EC_50_s). Efficacies (*E*_*max*_s) were determined as the % of the maximum contraction induced by carbachol (100 μM).

^a^ The lower mean *E*_*max*_ of the curve obtained in the strips pretreated with GRP may be related to the initial contraction evoked by GRP (10 nM), which was 6.7% ± 1.1% of the maximal contraction induced by CCh (*n* = 10).

## 4 Discussion

To date, most studies using isolated GI tissues to study the functions of GRP, NMB, and other BB_1_ and BB_2_ receptor agonists have examined their ability to cause tonic muscle contractions and/or increase the amplitude and frequency of spontaneous phasic contractions in various mammalian species, GI regions, and muscle layers [4]. The present study, using mouse stomach, is consistent with their findings. Thus, GRP, NMB, and BB induced concentration‐dependent contractions, with GRP and BB showing overlapping potencies (EC_50_s 63.1 and 43.4 nM, respectively) and efficacies (*E*_*max*_s 54.0 and 47.2, respectively). Lower EC_50_s are reported for the gastric fundus longitudinal muscle of both rat (GRP: 10–20 nM; BB: 1.3–6.5 nM [11, 31, 32]) and mouse (GRP_14‐27_: 7 nM [33]). This comparison suggests a greater sensitivity to BB and GRP in longitudinal versus circular muscle, although confirmation is required by studies in the same laboratory.

In our study, GRP was 7.4× more potent and ~40% more effective than NMB. The mean EC_50_ of NMB was 468.8 nM, ~1.8 times higher than that found in longitudinal muscle of rat gastric fundus (200–320 nM) [11, 32] and circular muscle of cat gastric fundus (260 nM) [34], and much higher than in longitudinal muscle of mouse gastric fundus (14.4 nM) [33]. However, in the latter study, NMB was only about half as potent as GRP_14–27_, and both had approximately the same efficacy [33]. Using the longitudinal muscle of rat gastric fundus, Regoli et al. [32] found that GRP was 10× more potent than NMB, similar to the present study using circular muscle of mouse gastric fundus. Overall, these small variations in relative potencies are unlikely to be explained by species differences in BB receptors, given the > 90% homology between mouse, rat, and human receptors [35]. However, they may indicate differences in efficiency of receptor coupling between species and muscle layers.

In the present study, the contractions evoked by GRP and NMB were not modified by the BB_1_ receptor antagonist PD168368 (0.1 μM), whereas they were inhibited by the BB_2_ receptor antagonist BW‐10 (1 μM). The 0.1 μM concentration of PD168368 was chosen because the selectivity for BB_1_ over BB_2_ receptors is not high (*K*_*i*_'s of PD168368 for rat BB_1_ and BB_2_ receptors are 39–45 nM and 1.3 μM, respectively, an ~30‐fold difference [25]). At 0.1 μM, ~2.5 times *K*_*i*_ for BB_1_ receptors and 8% *K*_*i*_ for BB_2_ receptors, the percentage occupancy of BB_1_ receptors by PD168368 should be sufficient to inhibit effects mediated by this receptor, while the percentage occupancy of BB_2_ receptors should be of little pharmacological significance. In the experiments of Ryan et al. [25], PD168368 (0.1 μM) inhibited by ~70%–80% the NMB‐stimulated increase in total [^3^H]inositol phosphates in BALB 3 T3 cells transfected with human BB_1_ receptors. On the contrary, the selectivity of BW‐10 for BB_2_ over BB_1_ receptors is high (*K*_*i*_'s for BB_2_ and BB_1_: 0.74 nM and > 10 μM, respectively [36]). Consequently, BW‐10 at 1 μM should occupy a high percentage of BB_2_ receptors. Indeed, BW‐10 at the much lower concentration of 10 nM, reduced BB‐induced gastrin release from isolated rat stomach by ~61% [37]. In our experiments, BW‐10 (1 μM) reduced the *E*_*max*_ of the concentration‐response curves for contractions induced by GRP and NMB (suggesting non‐competitive antagonism) and also increased the EC_50_ (suggesting competitive receptor antagonism). However, low concentrations of non‐competitive receptor antagonists can shift concentration‐response curves of agonists to the right when there are spare receptors, sufficient to produce a maximal response; higher concentrations reduce *E*_*max*_ when spare receptors are occupied. Thus, in summary, our data indicate that GRP and NMB act at BB_2_ receptors to contract circular smooth muscle of proximal mouse stomach, consistent with previous studies using proximal stomach from various mammalian species [11, 33, 34]. The contractions of longitudinal muscle of human and rat gastric fundus and circular muscle of cat gastric fundus evoked by BB [15, 31] or GRP_18–27_ [34] were not modified by TTX and/or atropine, suggesting direct action at the muscle.

The present study examined, for the first time, the effects of GRP on EFS‐evoked, nerve‐mediated contractions. These were abolished by TTX and greatly reduced by atropine (by 91.5%), suggesting predominant cholinergic involvement. The experiments were also conducted in the presence of inhibitors of adrenergic and nitrergic functions to optimize cholinergic activity. GRP induced a concentration‐dependent increase in these cholinergic‐mediated contractions. However, GRP did not modify contractions produced by bethanechol. Together, these data suggest that GRP facilitates the release of ACh that is induced by the arrival of the action potential at the nerve endings, without directly causing ACh release or influencing the response to direct activation of muscarinic receptors expressed by muscle cells and activated by bethanechol. This conclusion is consistent with other experiments, which demonstrate the absence of an inhibitory effect of TTX on bethanechol (rat stomach [38]) or GRP‐induced contractions (see above).

The prejunctional activity of GRP was inhibited by BW‐10, not PD168368, suggesting activation of BB_2_ receptors. However, the effects of BW‐10 were complex. Firstly, the *p*D_2_ was reduced without significantly changing the efficacy of GRP. This contrasted with the ability of BW‐10 to inhibit GRP‐induced muscle contractions, in which *E*_*max*_ was also reduced. One possibility is that the number of spare receptors expressed by enteric cholinergic neurons is greater than on muscle cells, and/or coupling of the receptors to intracellular effector pathways is more efficient for neuronal cells (notably the potency for the neuronal actions of GRP was also greater, compared with the effect on muscle). For either explanation, concentrations of BW‐10 greater than 1 μM would then be required to reduce the maximum ability of GRP to facilitate EFS‐induced contractions. The second complexity was that BW‐10 also inhibited EFS‐induced contractions by ~35%, probably by acting at a prejunctional site (BW‐10 (1 μM) did not inhibit contractions evoked by bethanechol). A possible explanation for this finding is that BW‐10 may have reduced EFS‐evoked contractions by antagonizing the ability of endogenous GRP to facilitate ACh release. Alternately, an additional, unknown property of BW‐10 inhibited cholinergic activity. Finally, it should be noted that the ability of BW‐10 to inhibit the effects of GRP was not big (an ~0.8 log unit shift in *p*D_2_). This may be explained by the limited percentage of BB_2_ receptor occupancy by BW‐10 at the concentration used. An alternate possibility that GRP activates BB_3_ receptors (resistant to antagonism by BW‐10) seems unlikely, given the low affinity of GRP for this receptor [39]. Thus, together, these observations suggest that to more fully explore the role of GRP within the ENS, further experiments are needed with BB_2_ receptor antagonists with higher affinity, structurally different, and perhaps more selective.

In our experiments examining neuronal activity, GRP also induced concentration‐dependent tonic contractions. Similarly, the TP receptor agonist U46619 produced concentration‐dependent tonic contractions, but did not increase EFS‐evoked contractions. These data further support the hypothesis that GRP facilitated ACh release by enteric cholinergic neurons. A role for GRP within the ENS is further suggested by the presence of GRP‐ and GRP_18–27_‐like immunoreactivity, and NMB‐like immunoreactivity in cell bodies of the myenteric plexus and fibers within muscle layers and mucosa [40, 41, 42, 43]. Binding sites for ^125^I‐labeled BB have also been demonstrated in the muscle layers and myenteric plexus (neuronal cell bodies and fibers) of dog stomach [8]. Finally, GRP and BB induce depolarization, increase excitability, and abolish post‐spike hyperpolarizing potentials, electrical effects similar to slow synaptic excitation, in AH/Type 2 myenteric neurons of guinea pig ileum [21], and GRP induced expression of Fos‐like immunoreactivity in myenteric neurons of rat gastric antrum [44].

In our work, GRP had a more potent ability to increase cholinergic‐mediated contractions (EC_50_ 14.7 nM), compared with its ability to cause muscle contraction (63.1 nM). Perhaps this indicates a preferred coupling of BB_2_ receptors to their effector mechanisms in myenteric neurons, relative to the receptors in smooth muscle. Thus, GRP‐releasing neurons could facilitate the release of ACh from excitatory motoneurons. This hypothesis is supported by studies reporting an important role for GRP in gastric emptying function. In one study, administration of anti‐BB monoclonal antibodies to dogs reduced the gastric emptying rate [6]. Additionally, the BB_2_ receptor antagonist BIM26226, administered intravenously to healthy volunteers, increased lag time and decreased gastric emptying rate measured by scintigraphy after ingestion of a solid meal containing ^99m^Tc [45]. Therefore, it could be hypothesized that GRP‐releasing neurons are activated throughout the duration of gastric emptying and that their ability to facilitate ACh release from excitatory motor neurons plays a physiological role.

In conclusion, the results of our study using mouse gastric fundus indicate that activation of BB_2_ receptors induces contraction of the circular muscle and facilitation of cholinergic‐mediated contractions, most likely by facilitating release of ACh from motor neurons. This supports the suggestion that GRP‐releasing neurons play a physiological role in the control of excitatory motor neurons in the proximal stomach. However, the physiological conditions under which this may occur and the relationship between the BB_2_ receptors on smooth muscle and enteric cholinergic neurons are unclear.

## Author Contributions

D.C., R.M., and G.J.S. conceived and designed the study. D.C. and R.M. performed the experimental work. D.C. acquired the data, and D.C., R.M., and G.J.S. analyzed and interpreted the data. D.C. drafted the first version of the manuscript, and D.C., R.M., and G.J.S. revised it critically for important intellectual content. D.C., R.M., and G.J.S. gave final approval of the version of the manuscript to be published and agreed to be accountable for all aspects of the work.

## Ethics Statement

The work described in this article was carried out in accordance with the UK Animals Scientific Procedures Act (ASPA) 1986. Cadaveric tissue collection did not require ASPA authority. Mice were killed humanely by cervical dislocation and exsanguination under ASPA Schedule 1 at Queen Mary University of London (UK Home Office License Ref XEDA0E7B1). The protocol and procedures employed were reviewed and approved by the institutional Animal Welfare Ethical Review Board for Queen Mary University of London, and personnel were trained and certified by the Named Training and Competency Officer and Named Animal Care and Welfare Officer.

## Conflicts of Interest

The authors declare no conflicts of interest.

## Data Availability

The data that support the findings of this study are available from the corresponding authors upon reasonable request.

## References

[1] A. Anastasi , V. Erspamer , and M. Bucci , “Isolation and Structure of Bombesin and Alytesin, 2 Analogous Active Peptides From the Skin of the European Amphibians Bombina and Alytes,” Experientia 27 (1971): 166–167, 10.1007/BF02145873. 5544731

[2] T. J. McDonald , H. Jörnvall , G. Nilsson , et al., “Characterization of a Gastrin Releasing Peptide From Porcine Non‐Antral Gastric Tissue,” Biochemical and Biophysical Research Communications 90 (1979): 227–233, 10.1016/0006-291x(79)91614-0. 496973

[3] N. Minamino , K. Kangawa , and H. Matsuo , “Neuromedin B: A Novel Bombesin‐Like Peptide Identified in Porcine Spinal Cord,” Biochemical and Biophysical Research Communications 114 (1983): 541–548, 10.1016/0006-291x(83)90814-8. 6882442

[4] I. Ramos‐Álvarez , P. Moreno , S. A. Mantey , et al., “Insights Into Bombesin Receptors and Ligands: Highlighting Recent Advances,” Peptides 72 (2015): 128–144, 10.1016/j.peptides.2015.04.026. 25976083 PMC4641779

[5] J. B. Furness , A. S. Miller , and M. Costa , “The Presence and Possible Roles of Bombesin‐Like Peptides in Enteric Neurons,” Annals of the New York Academy of Sciences 547 (1988): 76–82, 10.1111/j.1749-6632.1988.tb23877.x. 3071225

[6] M. E. Sunday , L. M. Kaplan , E. Motoyama , W. W. Chin , and E. R. Spindel , “Gastrin‐Releasing Peptide (Mammalian Bombesin) Gene Expression in Health and Disease,” Laboratory Investigation 59 (1988): 5–24. 2839735

[7] R. T. Jensen and T. W. Moody , “Bombesin‐Related Peptides,” in Handbook of Biologically Active Peptides, ed. A. Kastin (Elsevier Science & Technology, 2013), 1188–1196.

[8] S. R. Vigna , C. R. Mantyh , A. S. Giraud , A. H. Soll , J. H. Walsh , and P. W. Mantyh , “Localization of Specific Binding Sites for Bombesin in the Canine Gastrointestinal Tract,” Gastroenterology 93 (1987): 1287–1295, 10.1016/0016-5085(87)90257-5. 3678747

[9] T. H. Moran , T. W. Moody , A. M. Hostetler , P. H. Robinson , M. Goldrich , and P. R. McHugh , “Distribution of Bombesin Binding Sites in the Rat Gastrointestinal Tract,” Peptides 9 (1988): 643–649, 10.1016/0196-9781(88)90177-5. 2843836

[10] P. W. Mantyh , M. Catton , J. E. Maggio , and S. R. Vigna , “Alterations in Receptors for Sensory Neuropeptides in Human Inflammatory Bowel Disease,” in Advances in Experimental Medicine and Biology, vol. 298 (Springer US, 1991), 253–283, 10.1007/978-1-4899-0744-8_24. 1659149

[11] E. E. Ladenheim , K. A. Moore , C. F. Salorio , et al., “Characterization of Bombesin Binding Sites in the Rat Stomach,” European Journal of Pharmacology 319 (1997): 245–251, 10.1016/s0014-2999(96)00854-0. 9042597

[12] M. Rettenbacher and J. C. Reubi , “Localization and Characterization of Neuropeptide Receptors in Human Colon,” Naunyn‐Schmiedeberg's Archives of Pharmacology 364 (2001): 291–304, 10.1007/s002100100454. 11683516

[13] C. Porcher , A. Juhem , A. Peinnequin , and B. Bonaz , “Bombesin Receptor Subtype‐3 Is Expressed by the Enteric Nervous System and by Interstitial Cells of Cajal in the Rat Gastrointestinal Tract,” Cell and Tissue Research 320 (2005): 21–31. 15726424 10.1007/s00441-004-1032-1

[14] G. Bertaccini , “Peptides: Candidate Hormones,” in Mediators and Drugs in Gastrointestinal Motility II, ed. G. Bertaccini (Springer‐Verlag, 1982), 85–135.

[15] F. E. Lüdtke , B. Pogrzeba , K. Mandrek , G. Lepsien , T. Neufang , and K. Golenhofen , “Bombesin—The Most Stimulating Peptide of Human Gastric Smooth Muscle,” Digestive Diseases 9 (1991): 371–381. 1804577 10.1159/000171326

[16] C. Scarpignato , B. Micali , F. Vitulo , G. Zimbaro , and G. Bertaccini , “The Effect of Bombesin on Gastric Emptying of Solids in Man,” Peptides 2, no. Supplement 2 (1981): 199–203. 6892477 10.1016/0196-9781(81)90031-0

[17] C. Scarpignato , B. Micali , F. Vitulo , G. Zimbaro , and G. Bertaccini , “Inhibition of Gastric Emptying by Bombesin in Man,” Digestion 23 (1982): 128–131, 10.1159/000198702. 7095313

[18] G. Varga , R. M. Liehr , C. Scarpignato , and D. H. Coy , “Distinct Receptors Mediate Gastrin‐Releasing Peptide and Neuromedin B‐Induced Delay of Gastric Emptying of Liquids in Rats,” European Journal of Pharmacology 286 (1995): 109–112. 8566147 10.1016/0014-2999(95)00567-5

[19] J. G. Verbalis , M. J. McCann , and E. M. Stricker , “Species‐Specific Effects of Bombesin on Gastric Emptying and Neurohypophyseal Secretion,” Peptides 9 (1988): 1289–1293. 3247250 10.1016/0196-9781(88)90194-5

[20] E. E. Ladenheim , A. Wohn , W. O. White , G. J. Schwartz , and T. H. Moran , “Inhibition of Gastric Emptying by Bombesin‐Like Peptides Is Dependent Upon Cholecystokinin‐A Receptor Activation,” Regulatory Peptides 84 (1999): 101–106. 10535415 10.1016/s0167-0115(99)00078-6

[21] D. H. Zafirov , J. M. Palmer , P. R. Nemeth , and J. D. Wood , “Bombesin, Gastrin Releasing Peptide and Vasoactive Intestinal Peptide Excite Myenteric Neurons,” European Journal of Pharmacology 115 (1985): 103–107. 4043229 10.1016/0014-2999(85)90590-4

[22] Q. Jiang , O. Koldovsky , A. Bedrick , P. Pollack , and F. Porreca , “Bombesin Differentially Affects Gastric Emptying in Suckling, Weanling and Adult Rats,” Journal of Pharmacology and Experimental Therapeutics 257 (1991): 603–607, 10.1016/S0022-3565(25)24744-9. 2033507

[23] A. R. Chikh‐Issa , C. Scarpignato , M. Collinet , J. A. Chayvialle , and M. Vagne , “Dual Effect of Bombesin and Gastrin Releasing Peptide on Gastric Emptying in Conscious Cats,” Peptides 10 (1989): 281–287, 10.1016/0196-9781(89)90031-4. 2755871

[24] R. Makwana , J. Loy , M. Adebibe , K. Devalia , P. L. Andrews , and G. J. Sanger , “Copeptin, a Surrogate Marker of arginine^8^ Vasopressin, has no Ability to Modulate Human and Mouse Gastric Motility,” European Journal of Pharmacology 892 (2021): 173740. 33220268 10.1016/j.ejphar.2020.173740

[25] R. R. Ryan , T. Katsuno , S. A. Mantey , et al., “Comparative Pharmacology of the Nonpeptide Neuromedin B Receptor Antagonist PD 168368,” Journal of Pharmacology and Experimental Therapeutics 290 (1999): 1202–1211. 10454496

[26] N. González , S. A. Mantey , T. K. Pradhan , et al., “Characterization of Putative GRP‐ and NMB‐Receptor Antagonist's Interaction With Human Receptors,” Peptides 30 (2009): 1473–1486. 19463875 10.1016/j.peptides.2009.05.007 PMC2766550

[27] L.‐H. Wang , D. H. Coy , J. E. Taylor , et al., “Desmethionine Alkylamide Bombesin Analogues: A New Class of Bombesin Receptor Antagonists With Potent Antisecretory Activity in Pancreatic Acini and Antimitotic Activity in Swiss 3T3 Cells,” Biochemistry 29 (1990): 616–622. 1692477 10.1021/bi00455a004

[28] S. D. Harding , J. L. Sharman , E. Faccenda , et al., “The IUPHAR/BPS Guide to Pharmacology in 2018: Updates and Expansion to Encompass the New Guide to Immunopharmacology,” Nucleic Acids Research 46 (2018): D1091–D1106, 10.1093/nar/gkx1121. 29149325 PMC5753190

[29] S. P. H. Alexander , A. Christopoulos , A. P. Davenport , et al., “The Concise Guide to Pharmacology 2023/24: G Protein‐Coupled Receptors,” British Journal of Pharmacology 180, no. 2 (2023): S23–S144, 10.1111/bph.16177. 38123151 PMC13324819

[30] V. Ipavec , M. Martire , V. Barrese , M. Taglialatela , and D. Currò , “KV7 Channels Regulate Muscle Tone and Nonadrenergic Noncholinergic Relaxation of the Rat Gastric Fundus,” Pharmacological Research 64, no. 4 (2011): 397–409, 10.1016/j.phrs.2011.06.016. 21740972 PMC3178758

[31] F. Girard , H. Bachelard , S. St‐Pierre , and F. Rioux , “The Contractile Effect of Bombesin, Gastrin Releasing Peptide and Various Fragments in the Rat Stomach Strip,” European Journal of Pharmacology 102 (1984): 489–497, 10.1016/0014-2999(84)90570-3. 6489436

[32] D. Regoli , S. Dion , N. E. Rhaleb , G. Drapeau , N. Rouissi , and P. D'Orléans‐Juste , “Receptors for Neurokinins, Tachykinins, and Bombesin: A Pharmacological Study,” Annals of the New York Academy of Sciences 547 (1988): 158–173, 10.1111/j.1749-6632.1988.tb23884.x. 2853591

[33] H. Ohki‐Hamazaki , Y. Sakai , K. Kamata , et al., “Functional Properties of Two Bombesin‐Like Peptide Receptors Revealed by the Analysis of Mice Lacking Neuromedin B Receptor,” Journal of Neuroscience 19 (1999): 948–954, 10.1523/JNEUROSCI.19-03-00948. 9920658 PMC6782144

[34] E. A. Milusheva , N. I. Kortezova , Z. N. Mizhorkova , et al., “Role of Different Bombesin Receptor Subtypes Mediating Contractile Activity in Cat Upper Gastrointestinal Tract,” Peptides 19 (1998): 549–556, 10.1016/s0196-9781(97)00467-1. 9533644

[35] E. Giladi , S. R. Nagalla , and E. R. Spindel , “Molecular Cloning and Characterization of Receptors for the Mammalian Bombesin‐Like Peptides,” Journal of Molecular Neuroscience 4 (1993): 41–54. 8391296 10.1007/BF02736689

[36] R. T. Jensen , J. F. Battey , E. R. Spindel , R. V. Benya , and International Union of Pharmacology , “LXVIII. Mammalian Bombesin Receptors: Nomenclature, Distribution, Pharmacology, Signaling, and Functions in Normal and Disease States,” Pharmacological Reviews 60 (2008): 1–42, 10.1124/pr.107.07108. 18055507 PMC2517428

[37] P. Singh , Y. S. Guo , F. C. Kull , and J. J. Leban , “A Novel Bombesin Receptor Antagonist (2258U89), Potently Inhibits Bombesin Evoked Release of Gastrointestinal Hormones From Rats and Dogs, In Vitro and In Vivo,” Regulatory Peptides 40 (1992): 75–86, 10.1016/0167-0115(92)90085-9. 1332139

[38] H. F. Wrzos , T. Tandon , and A. Ouyang , “Mechanisms Mediating Cholinergic Antral Circular Smooth Muscle Contraction in Rats,” World Journal of Gastroenterology 10 (2004): 3292–3298, 10.3748/wjg.v10.i22.3292. 15484303 PMC4572298

[39] S. A. Mantey , H. C. Weber , E. Sainz , et al., “Discovery of a High Affinity Radioligand for the Human Orphan Receptor, Bombesin Receptor Subtype 3, Which Demonstrates That it has a Unique Pharmacology Compared With Other Mammalian Bombesin Receptors,” Journal of Biological Chemistry 272 (1997): 26062–26071, 10.1074/jbc.272.41.26062. 9325344

[40] G. J. Dockray , C. Vaillant , and J. H. Walsh , “The Neuronal Origin of Bombesin‐Like Immunoreactivity in the Rat Gastrointestinal Tract,” Neuroscience 4, no. 11 (1979): 1561–1568, 10.1016/0306-4522(79)90019-8. 390417

[41] R. Buffa , I. Solovieva , R. Fiocca , et al., “Localization of Bombesin and GRP (Gastrin Releasing Peptide) Sequences in Gut Nerves or Endocrine Cells,” Histochemistry 76 (1982): 457–467, 10.1007/BF00489901. 7166510

[42] G. L. Ferri , T. E. Adrian , M. A. Ghatei , et al., “Tissue Localization and Relative Distribution of Regulatory Peptides in Separated Layers From the Human Bowel,” Gastroenterology 84 (1983): 777–786. 6186565

[43] M. Namba , M. A. Ghatei , A. E. Bishop , et al., “Presence of Neuromedin B‐Like Immunoreactivity in the Brain and Gut of Rat and Guinea‐Pig,” Peptides 6, no. Suppl 3 (1985): 257–263, 10.1016/0196-9781(85)90383-3. 3913907

[44] M. C. Washington and A. I. Sayegh , “Gastrin Releasing Peptides Increase Fos‐Like Immunoreactivity in the Enteric Nervous System and the Dorsal Vagal Complex,” Peptides 32 (2011): 1600–1605, 10.1016/j.peptides.2011.06.023. 21745514

[45] L. P. Degen , F. Peng , A. Collet , et al., “Blockade of GRP Receptors Inhibits Gastric Emptying and Gallbladder Contraction but Accelerates Small Intestinal Transit,” Gastroenterology 120 (2001): 361–368, 10.1053/gast.2001.21174. 11159876

